# *Plasmodium falciparum* Multidrug Resistance Proteins (*pf*MRPs)

**DOI:** 10.3389/fphar.2021.759422

**Published:** 2021-11-01

**Authors:** José Pedro Gil, Cláudia Fançony

**Affiliations:** ^1^ Department of Microbiology, Tumor and Cell Biology, Karolinska Institutet, Stockholm, Sweden; ^2^ Faculty of Sciences, BioISI—Biosystems and Integrative Sciences Institute, University of Lisbon, Lisbon, Portugal; ^3^ Global Health and Tropical Medicine, Institute of Hygiene and Tropical Medicine, Nova University of Lisbon, Lisbon, Portugal; ^4^ Centro de Investigação em Saúde de Angola (CISA)/Instituto Nacional de Investigação em Saúde (INIS), Caxito, Angola

**Keywords:** malaria, *Plasmodium falciparum*, multidrug resistance, MRP, ABC protein

## Abstract

The capacity of the lethal *Plasmodium falciparum* parasite to develop resistance against anti-malarial drugs represents a central challenge in the global control and elimination of malaria. Historically, the action of drug transporters is known to play a pivotal role in the capacity of the parasite to evade drug action. MRPs (Multidrug Resistance Protein) are known in many phylogenetically diverse groups to be related to drug resistance by being able to handle a large range of substrates, including important endogenous substances as glutathione and its conjugates. *P. falciparum* MRPs are associated with in vivo and in vitro altered drug response, and might be important factors for the development of multi-drug resistance phenotypes, a latent possibility in the present, and future, combination therapy environment. Information on *P. falciparum* MRPs is scattered in the literature, with no specialized review available. We herein address this issue by reviewing the present state of knowledge.

## Introduction

*Plasmodium falciparum* drug resistance represents a major challenge for the global control of malaria. As in many other biological systems, a main strategy for evading drug action is to decrease the likelihood of contact between the antimalarials and their specific target by promoting decreases in drug concentration in the relevant compartment. This is particularly true in multidrug resistance (MDR) phenomena where this is achieved simultaneously to drugs with very different structure and modes of action.

Historically, transporter proteins have been known to play a pivotal role in the capacity of the parasite to evade antimalarial action. *P. falciparum* harbors a relatively small number of trans-membrane proteins (the “permeome”), with approximately 120 having been recognized through bioinformatics approaches (Martin et al*.*, 2005; Martin et al*.*, 2009). Among these, 16 members are considered to be putative ABC transporters (Koenderink et al., 2010). The P-glycoprotein homologue (Pgh1), evolutionary related with the mammalian cancer drug resistance associated P-glycoprotein (Pgp), was the first to be discovered, more then 30 years ago (Foote et al., 1989; Wilson et al., 1989). Pgh1 is the product of the *pfmdr1* (multidrug resistance 1) gene. *Pfmdr1* SNPs and/or increased copy number have been well studied as markers of parasite resistance to multiple antimalarials (Gil and Krishna, 2017). Less studied, but with the potential to be key MDR factors are the ABCC type of ABC proteins, also known as Multidrug Resistance Transporters (MRPs). MRPs have been observed in many phylogenetically diverse groups as broad range transporters, with substrates including important endogenous substances as glutathione and its conjugates, as well as a many drugs with diverse structures.

Since the global implementation of Artemisinin Combination Therapies (ACT), resistance to key combinations like artesunate-mefloquine and duhydroartemisin-piperaquine, have emerged (Price et al., 2006; Amato et al., 2017; Witkowski et al., 2017). Further, and of special concern, there are reports of significantly decreased efficacy of Artemether-Lumefantrine, the most globally used ACT. Indeed, several clinical trials in West Africa have registered PCR-corrected values below the well-established WHO 90% cure rate watermark for treatment policy change (WHO, 2006; Plucinski et al., 2015, 2017; Fançony et al., 2016; Dimbu et al., 2021; Gansané et al., 2021). Parasite MRPs might be important resistance factors in these developments. In parallel, a significant pipeline of new antimalarial is presently under development. A carry over of versatile multidrug resistant parasite populations nourished from the ACT era might have a significant impact in the useful lifespan of these new antimalarial, part of them being planned to be launched in combination with presently used antimalarials, like lumefantrine (e.g. KAF156/LUM), amodiaquine (e.g. AQ/methylene blue) and piperaquine (e.g. PPQ/Fosmidomycin).

Unfortunately, information about *P. falciparum* MRPs is widely scattered in the literature, with no review ever been produced gathering and interpreting the available data. The present article intends to timely fill this gap.

## *Plasmodium falciparum* Harbors Two Multidrug Resistance Proteins

The term “MRP” relates to a subgroup of generally large (>1200 aa) members of the ABC superfamily, mostly involved in drug efflux among eukaryotes, and in a few cases in signal transduction (e.g. mammalian SUR). They are present in a broad phylogenetic range of organisms, including many Vertebrae groups, plants, fungi, as well as unicellular parasites, like *Plasmodia* and *Leishmania* spp.

The first MRP-like protein was reported in a parasite, *Leishmania tarentolae* at the Nederlands Cancer Institute in Amsterdam by M. Ouellette, during the late 1980s (Ouelette et al., 1990). Isolated from amplified H circles present in methotrexate resistant parasites, the protein was initially referred as a “new P-glycoprotein”, i.e. an homolougue of the mammalian P-glicoprotein (Pgp), the most studied ABC transporter and the only drug resistance associated ABC protein at that time. The concept of a completely new group of ABC transporters, as well as the MRP designation, was later contributed by S. Cole and collaborators at Queens University, Kingston, Ontario. In the beginning of the 1990s, the cancer cell line H69AR was considered as carrying an atypical mechanism of resistance, as its high IC_50_ levels against doxorubicin were not associated with an expected overexpression of Pgp (Cole, 1990). A comparative transcriptional approach between the sensitive progenitor H69 cell line with the H69AR derived doxorubicin resistant one revealed the presence of one mRNA *ca*. 100–200 fold more prevalent in the latter. The sequence analysis revealed a new ABC protein coding gene, designated *MRP* (presently *ABCC1*) (Cole et al., 1992), the formal founding member of the large ABCC subfamily. MRPs are related with xenobiotic handling, including many therapeutic drugs, in a large range of organisms. Contrarily to Pgp-like transporters, MRPs are capable of handling hydrophilic phase II metabolites, while still being able to handle non-conjugated compounds, frequently through co-transport with reduced glutathione (GSH) (Rappa et al., 1997).

**FIGURE 1:**
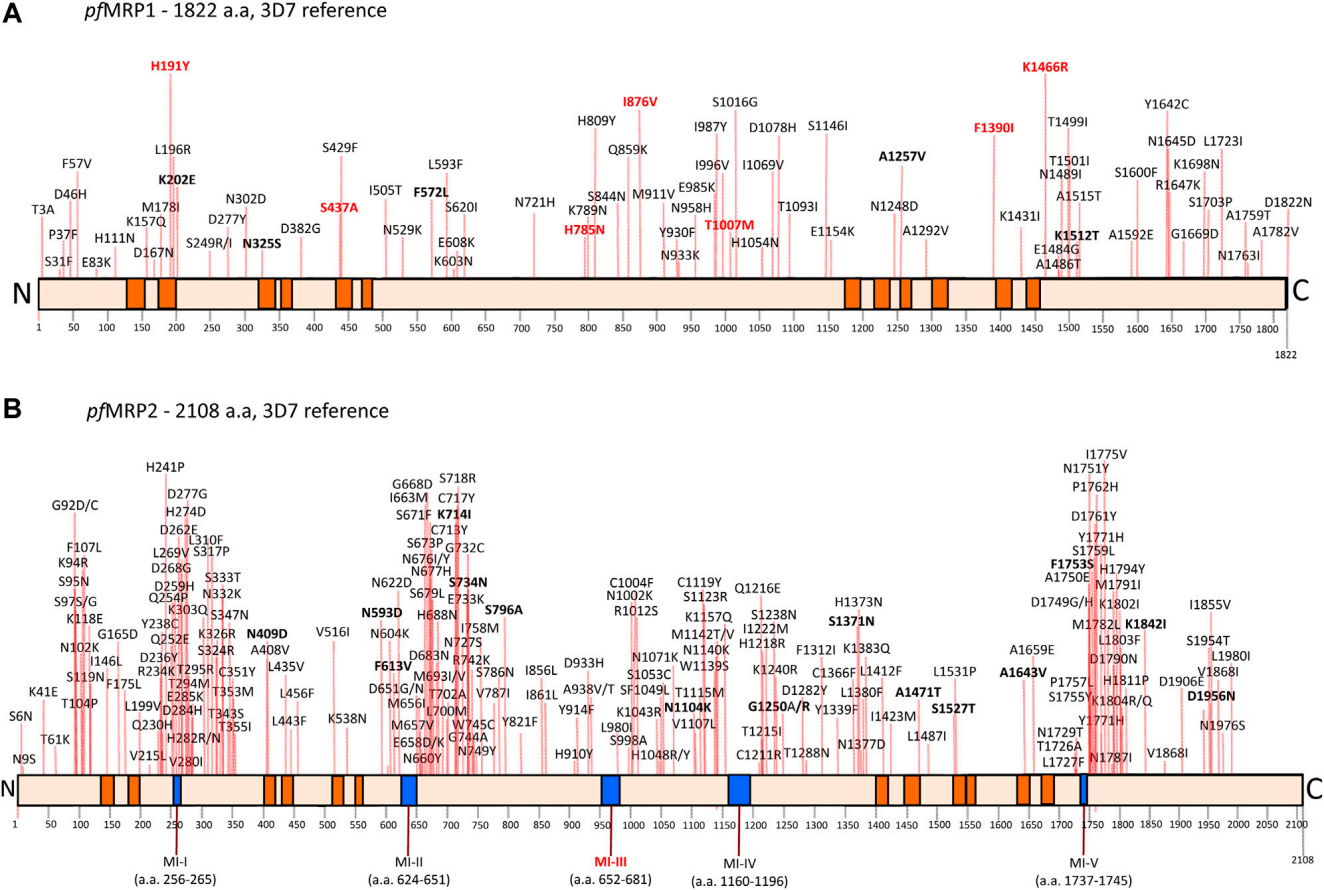
Compilation of non-synonymous SNPs in *pf*MRP1 **(A)** and *pf*MRP2 **(B)**. This was based on MalariaGen and the article list of bibliographic references. SNPs in bold correspond to alleles with frequencies above 1%, according to the MalariaGen Community Project. In bold red, SNPs that have been reported as associated with parasite drug response (see main text and Table 1). Boxes in orange denote transmembrane domains (Dahlstrom et al., 2009; Veiga et al., 2014). Blue boxes in the *pf*MRP2 figure correspond to micro-indel (MI) regions comprising variable tandem repeat regions (Veiga et al., 2014). MI-III has been observed to be associated with lumefantrine response (Okombo et al., 2013), being as such also marked in bold red. To note the trend for SNP hotspots in *pf*MRP2.

In terms of cell physiology, MRPs are frequently major oxidized glutathione (GSSG) transporters, linking them with the complex network of intracellular REDOX stress management. Such function is in turn consistent with the significant stress associated to the parasite intra-erythocytic cycle, to a large extent due to haemoglobin catabolism (Bozdech and Ginsburg, 2004). Accordingly, the first clear indication of MRPs presence in *P.falciparum* was contributed by H. Ginsburg’s group at the Hebrew University in Jerusalem. Upon the observation that GSSG concentrations inside the parasite were significantly lower than in the host RBC, the authors proposed that “it may be that the parasite membrane contains a specific ATP-driven GSSG pump” (Atamna and Ginsburg, 1997), which would be later functionally connected with a highly active glutathione metabolism (Ayi et al., 1998). The isolation of MRP coding genes in *P. falciparum* was preliminarily reported 3 years later, through an ABC signature targeting degenerate primer approach (Gil et al., 2000), revealing two intron-less genes, provisionally referred as *pfmrp1* and *pfmrp2*, later identified by the *Pf* genome sequencing project as *PF3D7_0112200* (previously, PFA0590w) and *PF3D7_1229100* (previously, PFL1410c), located at chromosome 1 and 12, respectively. Both code for ABC-transporter canonical 12 intra-membrane domains (Figure 1). Further characterization of *pf*MRP1, confirmed its size (1812 a.a., 3D7 reference genome, 210–215 kDa) and homology with several members of the MRP group (Kouklozas et al., 2004). *pf*MRP1 is present in every sexual and asexual blood stages of the parasite life cycle (Raj et al., 2009). The protein is essentially located in the plasma membrane (Kaviche et al., 2009; Koenderink et al., 2010; Rijpma et al., 2016) with some sporadic signals of its presence in internal sites, likely to be related with membrane turn-over processes during the asexual cycle (Kouklozas et al., 2004), or possibly associated with specific processes during the late gametocyte stages. In liver stages its localization is less clear (Rijpma et al., 2016).

As for the less studied *pf*MRP2, which share 41% primary sequence identity with *pf*MRP1, it represents a larger protein (ca. 2120 a.a., 3D7 reference genome), also localized in the parasite plasma membrane (Kaviche et al., 2009; Koenderink et al., 2010), albeit more internal localizations have been proposed, particularly in the later stages of the intra-RBC (Mok et al., 2014).

The confirmation of the existence of two MRP-like proteins coded in the *P. falciparum* genome, as compared with the typical presence of only one in rodent *Plasmodia* (Gonzalez-Pons et al., 2009; Koenderink et al., 2010) has been supported upon a number of detailed in silico data mining works (Bozdech and Ginsburg, 2004; Kirk et al., 2005; Martin et al., 2005, 2009; Kaviche et al., 2009).

## *pf*MRPs—Potential Natural Roles in the Parasite Physiology

The transcriptional activity of *pfmrp1* and *pfmrp2* along the intra-erythrocyte cycle has been analyzed, showing significantly different patterns for each gene (Bozdech et al., 2003; Bozdech and Ginsburg, 2004; Nogueira et al., 2010; Veiga et al., 2010). *pfmrp1* has its peak of expression in the trophozoite stage, *ca*. 28 h post-invasion coherent with a putative GSSG efflux function of the protein, as this phase is the most active in the digestion of hemoglobin, and its associated oxidative stress. Strategically, it coincides with the peak expression of glutathione synthetase, further supporting a probable role in REDOX stress management associated with the process of hemoglobin digestion. *Pfmrp2* on the other hand has its maximal expression still in the ring stage, with a maximum at around 21 h after invasion, suggesting the possibility of these proteins having different natural functions (Bozdech and Ginsburg, 2004; Veiga et al., 2010; Mok et al., 2014).

Whatever *pf*MRP1 and *pf*MRP2 roles are in *P. falciparum* physiology, they can be dispensable for the intra-erythocytic cycle. Gene editing experiments have shown that the full deletion of *pfmrp1* in W2 (Raj et al., 2009) and NF-54 (the strain from which 3D7 clone was obtained) (Rijpma et al., 2016; Rijpma et al., 2016), as well as the *pfmrp2* ablation in the latter, renders viable parasites—at least at *in vitro* conditions, and with these particular clones.

W2/MRP∆ showed clear evidence for fitness cost upon *pf*MRP1 absence (Raj et al., 2009), namely an incapacity for the cultures to reach parasitemias >5%, due to a lower efficiency of the resulting merozoites to re-invade new RBCs. Additionally, W2/MRP∆ tended to develop gametocytes faster then WT W2, an effect interpreted as a response to a decreased capacity to manage environmental stress due to *pf*MRP1 absence, leading to an earlier triggering of the switch from asexual to sexual stages. These effects were not observed for the modified NF54 clone, even when deleting each or both *pfmrp1* and *2* (*Pf*∆mrp1∆mrp2) (Rijpma et al., 2016). Nevertheless, oocyte production is significantly reduced in this double mutant, albeit the resulting sporozoytes demonstrated normal gliding and hepatocyte transverse capabilities (Rijpma et al., 2016). These differences between clones suggest that the degree of impact of *pf*MRP1 and 2 functions in these blood stages is likely to be dependent of the genomic/metabolomic environment where these proteins are embedded. Some genomes (like NF54) might include the capacity of compensatory mechanisms, including other transporters, apparently not available in the W2 clone. The question is anyway still open, as emphasysed by the *in vitro* observation that 3D7 is sensitive to N-Benzyloxycarbony-S-(2,4-dinitrophenyl)-glutathione diesters, a class of specific MRP inhibitor proposed as proof of concept compounds for defining MRPs as valuable antimalarial targets (Daunes and D'Silva, 2018).

As for *pf*MRP2, albeit not essential for the intra-erythorcytic cycle, or sexual development (at least in the NF54 context), it has shown to be a key factor during the liver stage of the parasite (Rijpma et al., 2016). Parasites devoid of *pf*MRP2 are capable of invading hepatocytes and proceed with a normal intra-cellular development. They are anyway unable to form mature merozoites pointing for *pf*MRP2 as a critical component of *P. falciparum* liver schizont formation.

## Sequence Diversity

Presently approximately 80 non–synonymous *pf*MRP1 SNPs are known (Figure 1), the majority of them present at very low (<0.1%) global frequencies (data mainly provided through the MalariaGen project). The global distribution of SNPs is variable, with some positions being widely distributed, like the frequently linked Y191H/A437S (Mu et al., 2003; Ursing et al., 2006; Pirahmadi et al., 2013), as well as I876V (Dahlstrom et al., 2009; Gupta et al., 2014; Zhao et al., 2019; Al-Ruhmi et al., 2020), which seems to have been circulating at least since pre-chloroquine times (de Dios et al., 2019). Others are clustered in some regions, like the 1466 position, frequent in East Africa (Dahlstrom et al., 2009; 2009a), but rare in the Western coast (Otienoburu et al., 2016; Jovel et al., 2017), and found absent in studies in Asia (Veiga et al., 2011; Gupta et al., 2014; Phompradit et al., 2014) as well as in the Americas (Dahlstrom et al., 2009). *Pfmrp1* gene copy number variation has been observed *in vitro* (Bopp et al., 2013) and is also likely to exist in the field, as recently suggested through deep analysis of next generation sequencing archive data from West African parasites (Yini et al., 2018). Further studies are needed to confirm these bioinformatics-based searches.

With over 200 SNPs already identified^1^ (Veiga et al., 2014), *pf*MRP2 seems to harbor significantly more polymorphism then *pf*MRP1 (Figure 1). Consistent with this diversity, sequencing the *pfmrp2* gene in a set of 46 SE Asian adapted parasites from the Thai-Burma border revealed a number of micro-indel (MI) regions (MI I-V), giving rise to proteins with significantly different lengths (Veiga et al., 2014).

## *pf*MRPs and Antimalarial Drug Response

### Chloroquine

Early support for the involvement of *P. falciparum* MRPs in malaria drug resistance came from one of the first re-sequencing projects after unveiling *P. falciparum*’*s* genome (Mu et al*.*, 2003). Upon the ORF sequencing of 49 putative transporter genes in 97 culture-adapted parasites from widely diverse geographical origins, associations with chloroquine (and quinine, see below) IC_50_ levels were searched. Positive associations were found between *pfmrp1* (originally termed “G2”) H191Y and S437A SNPs and increased CQ IC_50_’s [(^3^H) hypoxanthine uptake methods, 48 h (Desjardin et al., 1979)] Such results were consistent with the fact that chloroquine (CQ) is an MRP1 substrate in human cells (Vezmar and Georges, 1998), and a potential factor in modulating the use of this drug for arthritis management (Oerlemans et al., 2006).

A re-evaluation of these *pfmrp1* SNPs was subsequently performed in a set of 107 ex-vivo characterized infections from a single hospital in the Thai-Myanmar border during a short period of time, avoiding biasing from comparing parasites derived from populations with different genetic structures (Anderson et al., 2005). The analysis did not reproduce the CQ vs. H191Y/S437A associations, albeit it has to be noted that both *pfmrp1* polymorphisms were fixed (Y191: 100%) or near fixed (A437: 86%) in the region, severely limiting the power of the study. Also, the remaining of the gene ORF was not investigated, not ruling out the potential involvement of other SNPs, as later investigated (e.g. Veiga et al., 2011; Bai et al., 2018). Such SNP associations with CQ IC_50_s [HRP2 double-site Sandwich linked immunosorbent assay, 72 h (Noedl et al., 2005)] were not observed in the analysis of a set of 48 SE Asian parasites from the same Thai-Myanmar Western border region, where the full *pfmrp1* ORF was characterized (Veiga et al., 2011). Differently from the (Mu et al., 2003) collection of analyzed strains, all these SE Asian isolates were highly resistant to CQ. This might have overshadowed a potential secondary *pf*MRP1 contribution, likely to be only detectable through a much larger trial. A more recent study in the Mae Sot region found a higher prevalence of the resistance associated 191Y allele (13%) among 119 adapted parasites, having actually reported a significant association with CQ IC_50_s Phompradit et al., 2014). In another study performed with culture adapted parasites now from the China-Myanar border during the 2007–2012, the T1007M polymorphism was associated with higher CQ IC_50_s (Bai et al., 2018).

By mainly using parasites adapted from returning travellers to France, Pradines and collaborators published a series of reports analyzing relatively small sets of strains (n = *ca*. 20–23) from different geographical origin and range of CQ sensitivities (Henry et al., 2008; Henry et al., 2009) (Table 1). Contrarily to the reports focused on SE Asia, the authors repeatidly observed associations of the H191Y and S437A polymorphisms and increased CQ IC_50_ values in these sets of parasites of heterologous origin (it is important to note that between reports a significant degree of overlap exists between the sets of strains used). These studies, supportive of the aforementioned initial observations (Mu et al., 2003) were limited to the analysis of these two SNPs, opening the possibility of the latter to be in linkage with yet other participating polymorphisms in the *pfmrp1* ORF. Consistent with this view, the I876V SNP—previously connected with *in vivo* artemether-lumefantrine (AL) response (Dahlstrom et al., 2009) (see below)—was seen associated with increased CQ IC_50_s (SYBR green based fluorescence assay, 72 h) (Meng et al., 2010) in 63 *P. falciparum* adapted parasites from the Northern Thai-Chinese border (Hao et al., 2013). The fully sequencing of the *pfmrp1* coding region did not unveil other associations, including with the H191Y and S437A SNPs, which were as prevalent as I876V. The global prevalence of the latter SNP was speculated to be a remnant of the worldwide spread of CQ resistance during the XX Century (Hao et al., 2013). A later, smaller study, found an additional association with the I1390 allele (Gupta et al., 2014), which has not been previously detected (Veiga et al., 2011).

**TABLE 1:** *pf*MRP Sequence variation associated with *P. falciparum* antimalarial response.

SNP and drugs	Drug-response associations
pfMRP1—H191Y/S437A^a^	
CQ	Y/A: Increased IC_50_ [1–9]
DEAQ/ASAQ	Y/A: Increased IC_50_ [2, 3, 6–8]
MQ	Y/A: Increased IC_50_ [10]
QN	Y/A: Increased IC_50_ [1–8]
pfMRP1—H785N	
PPQ	N: Increased IC_50_ [11]
LUM	N: Increased IC_50_ [11]
pfMRP1—I876V	
CQ	V: Increased IC_50_ [12]
AL	I: Significant *in vivo* I876 selection by treatment [13]
MQ	V: Increased IC_50_ [10]
DHA-PPQ	V: Delayed parasite clearance [14]
pfMRP1—T1007M	
CQ	M: Increased IC_50_ [11]
PPQ	M: Increased IC_50_ [11]
pfMRP1—F1390I	
CQ	I: Increased IC_50_ [15]
QN	I: Increased IC_50_ [9]
MQ	F: Increased IC_50_ [16]
LUM	F: Increased IC_50_ [16]
Artemisinin/DHA	F: Increased IC_50_ [11, 16]
pfMRP1—K1466R	
SP	K: Selection upon SP treatment [17]
pfMRP2—MI-III	
LUM	7-DNNNTS/NNNNTS: Increased IC_50_ [18]

References: [1] Mu et al., 2003; [2] Henry et al., 2009; [3] Henry et al., 2008; [4] Parquet et al., 2009; [5] Parquet et al., 2010; [6] Pradines et al., 2010; [7] Briolant et al., 2010; [8] Pascual et al., 2011; [9] Phompradit et al., 2014; [10] Phompradit et al., 2014a; [11] Bai et al., 2018; [12] Hao et al., 2013; [13] Dahlström et al., 2009; [14] Lo et al., 2016; [15] Gupta et al., 2014; [16] Veiga et al*.*, 2011; [17] Dahlstrom et al., 2009a; [18] Okombo et al., 2013.

a Frequently linked.

These associations between CQ response and *pf*MRP1 are supportive of previous genetic modification works in W2, reporting a significant decrease in IC_50_’s (72-h incubation essays) of ca. 1.6 fold (∼160–∼100 nM) *pfmrp1* excision (Raj et al., 2009). The authors further explored the possibility of these differences to be related with the capacity of *pf*MRP1 to transport CQ. This was performed through 6-point drug accumulation essays, using radioactive labeled (^3^H) chloroquine for a period of 1 hour. Scintilation rate measures of the intact parasites showed a significant increase in CQ accumulation in the *pfmrp1* knocked-out parasite. At the final time point of the experiment (60 min), the difference in accumulation was *ca*. 2 fold.

Somewhat surprisingly, this sensitization effect was not registered by Rijpma et al. (2016) when deleting *pfmrp1* (and *pfmrp2*) in the NF54 strain. In fact, and as referred in more detail below, the ablation of these genes essentially did not significantly influence the NF54 *in vitro* IC_50_s against quinolone drugs—with the possible exception of MQ—and artemisinins. The reasons behind these marked differences are not clear. As previously referred in terms of fitness effects, they might be related to the different genomic context between the South East Asian W2 and the NF54 East African strains.

CQ was recently demonstrated *in vitro* to directly interact with *pf*MRP1. Using a CQ 7-nitrobenz-2-oxa-1,3-diazole (NBD)-labeled fluorescent derivative, Woodland and collaborators detected significant fluorescence in the parasite plasma membrane. A drug-labeled affinity matrix based approach subsequently identified a 200–250 Kd plasma membrane associated protein. Through mass spectrometry-based proteomics, this protein was further shown to be *p*fMRP1 (Woodland et al., 2018), supporting the concept of CQ as a substrate for this ABC transporter, and consequently, a factor in the parasite response.

As for *pf*MRP2, a suggested trend for association between the synonymous SNP t3414c and chloroquine IC_50_s (660 vs. 564 nM) has been reported (Veiga et al., 2014). Any contribution of ORF polymorphism of this transporter in CQ response is likely to be minor. The question remains on the importance of non-coding SNPs located in the putative 5′regulatory regions of the gene, taking in consideration the implications of transcriptional control in this gene and drug response (Mok et al., 2014).

### Desethylamodiaquine

As for amodiaquine’s clinically relevant metabolite desethylamodiaquine (DEAQ), less data is available (Table 1). The *pf*MRP1 H191Y/S437A SNPs have been reported to be significantly linked with increased DEAQ IC_50_s in 15 adapted parasites (Henry et al., 2008), a result subsequently supported through the same approach (Parquet et al., 2009; Briolant et al., 2010; Pradines et al., 2010; Pascual et al., 2011). Such an association was not previously observed in a set of 15 South American parasite strains (Western Colombia) (Echeverry et al., 2007) where, albeit a large range of DEAQ (and CQ) responses (DEAQ IC_50_’s *ca.* 40–350 nM, CQ IC_50_’s *ca.* 115–415 nM), all *pfmrp1* ORFs were 3D7-like (“wild type”) (Dahlstrom et al., 2009).

A highly sensitive approach for detecting the involvement of a particular gene in antimalarial drug response is the monitoring of gene polymorphism selection upon antimalarial therapy. In general there are very few *in vivo* studies investigating this treatment driven selection of *pfmrp* SNPs among recurrent infections. To our knowledge, no such analysis has been performed in CQ or DHA-PPQ efficacy trials. As for AQ/DEAQ, an early report studying the H191Y/S437A SNPs in an AQ monotherapy study in Kenya did not show signs of selection (Holmgren et al., 2006). It must be anyway stressed that the low prevalence of both mutant alleles (<10%) and the small size of the study (81 patients), severely limited its power. Nevertheless, a larger follow-up analysis of two ASAQ clinical trials in Zanzibar and mainland Tanzania did not show any signs of H191Y/S437A selection (Dahlstrom et al., 2009), supporting the previous observations in Kenya. The subsequent analysis of an effectiveness study in Benin could not reach conclusions concerning the I876V SNPs due to rarity of the mutant alleles, while K1466R did not show trends of selection, or associations with DEAQ ex-vivo IC_50_s (Dahlstrom et al., 2014). Another study, now an efficacy trial in Liberia confirmed the low prevalence of the 876V (<1%) and 1466R (ca. 2.5%) alleles, not allowing the drawing of conclusions (Otienoburu et al., 2016). The available data prompts the conclusion that *pfmrp1* is at most a secondary contributor for AQ/DEAQ resistance.

### Quinine

As with CQ, (Mu et al., 2003) reported significant positive associations between the *pfmrp1* (“G2”) 191Y and 437A alleles and increased IC_50_ values. This result was later repeatedly confirmed in smaller collections of parasite strains using similar isotopic *in vitro* tests (Henry et al., 2008, 2009; Parquet et al., 2009; Parquet et al., 2010; Pradines et al., 2010; Briolant et al., 2010; Pascual et al., 2011) (Table 1). The importance of the 191 and 437 a.a. status with QN response field samples was not possible to be robustly tested by Anderson et al. (2005) in a large set (>200, 1998–2003 period) of ex-vivo characterized SE Asian parasites, due to the rarity of these polymorphisms found in that study, as previously referred. A subsequent study, also performed in the Mae Sot region (2006–2009 period) in 119 culture-adapted parasites extended the number of a.a. positions under scrutinity, with a significant association found between QN IC_50_s and F1390I (F1390: 238 nM, 1390I: 190 nM) (Phompradit et al., 2014).

### Piperaquine

The whole deletion of *pfmrp1* in the context of the W2 clone (W2/MRP∆) gave rise to a 1.8 fold (∼140–∼80 nM) decrease in IC_50_ (72 h exposure) to piperaquine. This is consistent with data where the uricosuric drug probenecid (PBN) has been shown to be able to sensitize *P. falciparum* to this antimalarial (Masseno et al., 2009), albeit it is important to note the relatively unspecificity of this reversal agent, which might be influencing other transport systems. The *pfmrp1* 191 and 437 SNPs were not associated with *in vitro* sensitivity in 23 parasite strains (Briolant et al., 2010), a result supported by Hao et al. (2013) when analyzing 63 strains from Northern Myanmar (Table 1). In the same region, a subsequent study found a significant increase in PPQ IC_50_s among *pfmrp1* 1007M and 785N carrying parasites (Bai et al., 2018), reinforcing the view of the need of full ORF sequences in order to more robustely unveil valuable associations with this gene.

### Aminoalcohol Quinolines: Lumefantrine and Mefloquine

Lumefantrine (LUM) is part of the most used antimalarial worldwide, in combination with artemether. Two independent Coartem^®^ (Artemether-Lumefantrine) clinical comparative efficacy trials in East Africa documented a significant selection of the *pfmrp1* 876I allele among recurrent parasites (frequencies of 11% *vs* 2.6%, respectively) (Dahlström et al., 2009). The I876V SNP is immediately adjacent to the Walker A/NBD1 (Figure 1). In silico analysis of the I→V change have suggested that even though the structural differences between these two a.a. are small, it may affect the rate of the ATP hydrolysis cycle of this ABC transporter (Dahlström et al., 2009). The *pfmrp1* 876I association was specific for Coartem^®^ - no significant selection was detected concerning the comparator chemotherapies, ASAQ as previously referred, and SP.

Mefloquine sensitivity was not changed by *pfmrp1* deletion in the W2 strain (Raj et al., 2009). These results are somewhat consistent with the *in vitro* data by Wurtz et al. (2010), where no significant associations were found between the 191 and 437 SNPs and MQ IC_50_s (n = 21 strains). On the other hand, in NF54, The removal of *pf*MRP1 led to a significant 2-fold increase in IC_50_ values (121 *vs* 241 nM) (Rijpma et al., 2016). Intrigingly, the deletion of *pf*MRP2 in this strain leads to an identical outcome (IC_50_ = 242 nM), and a similar one when both are missing (IC_50_ = 272 nM). This lack of effect reinforcement is somewhat unexpected, possibly meaning that whatever influence of the MRP system in MQ response, it reaches its maximum outcome with one of the proteins missing.

In South East Asia, *pf*MRP1 F1390I has been associated with significant changes in IC_50_ values (72 h exposure) in a set of 48 culture-adapted parasites from this region (Veiga et al*.*, 2011). F1390 carriers had significantly increased IC_50_s for both mefloquine (*ca*. 122 nM vs. 37 nm) and lumefantrine (16 nM vs. 6 nM). This 3’ ORF region polymorphism, frequent in the SE Asian regions (Nyunt et al., 2015, 2017), codes for an amino acid position located at—or at least in close interaction—with TM11. Assuming TM as part of the internal lining of the tranporter interacting with the drugs, it is possible that mutations in these regions (like the 191 and 437 a.a.) will directly participate in the transport process (Table 1). In 43 culture-adapted parasites from Burmanese patients, a significant association between the 191Y, 437A and 876V alleles and increased mefloquine IC50′s was reported, but no link with the F1390I SNP (Phompradit et al., 2014a). In another *in vitro* study from the northern Miyanmar-China region, the H785N SNP was associated with significant changes in LUM IC_50_s (Bai et al., 2018).

Considering the documented lower Artemether-Lumefantrine efficacy in these regions (Price et al., 2006), it is conceivable that *pfmrp1* genes harbouring this variant might constitute an additional contributing resistance factor, along with 86N carrying *pfmdr1* duplications (Calçada et al., 2020).

Concerning *pf*MRP2, IC_50_ determination (48 h exposure, [^3^H]hypoxanthine incorporation) of a selected set of culture adapted parasites (*n* = 67) pointed for a significant association between the presence of seven copies of ORF-located short repeats [DNNNTS/NNNNTS, indel region III (Veiga et al., 2014) (Figure 1)] and IC_50_ for lumefantrine (Okombo et al., 2013). Such associations were not seen among SE Asian culture adapted parasites (Veiga et al., 2014).

### Artemisinins

When considering each of the two MRPs independently, (Rijpma et al., 2016) did not documented changes in di-hydroartemisinin sensitivity in NF54, although a suggestive trend was noticed of less sensitivity to DHA when *pfmrp1* and *pfmrp2* were both ablated (43 vs. 134 nM). Again this comes in contrast with the reported for W2, where the deletion of *pfmrp1* led to a prompt sensitization to artemisinin (from 11 to 5 nM), not observed in NF54. Concerning sequence variation, the status of the previously mentioned *pf*MRP1 F1390I SNP was shown to influence the parasite *in vitro* response (72 h exposure), of both artesunate and DHA (Veiga et al., 2011), the F1390 allele being associated with increased IC_50_s (artemisinin: 9 vs 3 nM; DHA: 1.5 vs. 0.9 nM). This initial observation was supported in a larger set of SE Asian parasites (Bai et al., 2018). The sensitivity pattern associated to the presence of F1390I is operationally worrying, as it was seen to simultaneously increase parasite resistance against the two available amino-alcohol quinolone based ACTs, mefloquine-artesunate and lumefantrine-artemether. In sub-Saharan Africa, where Artemether-Lumefantrine represents the first line treatment in the majority of the national malaria control programs, the F1390 allele is fortunately still unusual (<1%), contrarily to Asia, where it can reach >50% in Indochina (Dahlstrom et al., 2009)^2^.

In Nigeria, a field ex-vivo study attempted to find a link between the *pfmrp1* S437A polymorphism and response to artemether (Bustamante et al., 2012), an important question considering the importance of AL in the continent. Unfortunately, the rarity of this allele (3% frequency in this study) precluded any meaningful conclusions. In terms of *in vivo* approach, ACT day-3 surveillance trials performed in the East and Western borders of Myanmar (Chin and Kayen provinces, respectively) in 2013 did not find associations between D3 positivity and the seven *pfmrp1* non-synonymous SNPs detected (Nyunt et al., 2015), not supporting an involvement of this gene in the parasite response to artemisinin type compounds (ART). In contrast, a significant link between the 876V allele and delayed parasite clearance was found in a study conducted among 63 patients in Laiza, Myanmar, treated with DHA-PPQ during 2011–2013 (Lo et al., 2016).

Due to the well-known ART pleiotropic effects and the complex architecture of associated resistance phenotypes, it is likely that other factors—including additional drug transporters—might be involved. The latter is to a certain extent supported by the recent demonstration that the ART-resistance kelch13 mutations lead to a rewiring of the parasite physiology (Mok et al., 2021). Finally, it is important to refer that concerning *in vitro* phenotype characterization, classical IC_50_s have been considered insufficient for a proper response profiling to this type of antimalarials (Davis et al., 2020), as they do not generally reflect variation in the main characteristic of artemisinin (partial) resistance, parasite clearance dynamics.

### The Particular Case of Folate and Antifolate Drugs

*P. falciparum* resistance to antifolate drugs, as pyrimethamine and sulfadoxine, is centrally associated with mutations in the parasite endogenous folate pathway, the metabolic target for these drugs. Here, mutations in the *pfdhfr* (dihydrofolate reductase) and *pfdhps* (dihydropteroate synthase) genes are critical (Warhurst, 2002), as well as increased copy number events in the GTP cyclohydrolase 1 (*pfgch1*) gene, coding for the first enzyme of the pathway (Heinberg and Kirkman, 2015). Additional factors have nevertheless been long proposed, in particular with the involvement of transport systems (Wang et al., 1997).

#### The Folate Pool Factor

Concentration of folate influences the efficacy of anti-folate drugs (Wang et al., 1997; Wang et al., 1999), as an increase in its intra-cellular pool will compete with these agents action, and partially rescue the parasite. Fluctuations in this pool are dependent on both, the parasite endogenous folate biosynthesis, and its efficient salvage pathway aimed on extracting this factor from the host (Nzila et al., 2004). The latter is acted through two identified folate importers, pfFT1 and 2 (folate transporter 1 and 2) (Salcedo-Sora et al., 2011), their function having been probed by the transporter inhibitor probenecid (PBN), *in vitro* exposure to which leads to a decreased parasite uptake of radiolabeled folic acid (Nzila et al., 2003).

A third factor might be *pf*MRP1. Mammalian MRPs are well known as being able to transport folates and antifolate drugs. Consistent with a potential involvement of these proteins, the *pfmrp1* R1466K SNP has been reported to be selected upon Sulfadoxine-Pyrimethamine antimalarial treatment in East Africa (Dahlstrom et al., 2009a). This data is interpretable as evidence of a *pf*MRP1 folate efflux activity, the 1466K allele leading to a less efficient transport, hence aiding the maintenance of higher folate levels, and consequently parasite reduced pyrimethamine/sulfadoxine sensitivity. This hypothesis gained support by experiments with genetically edited NF54 parasites with deleted *pfmrp* genes. Through an untargeted metabolomics screening of erythocyte lysates, folate was identified as a major *pf*MRP1 substrate candidate (Rijpma et al., 2016a).

Anyway, the scenario of *pf*MRP1 involvement in anti-folate drug response is far from clear. The first point is the fact that PBN is a leaky factor, being also a MRP inhibitor. It would be expected that its use should block the folate efflux out of the parasite, an effect that would lead to increased resistance, not sensitivity. This lack of signal might be primarily explained by a much smaller *pf*MRP1 contribution as compared with the actions of *pf*FT1 and 2 (Salcedo-Sora et al., 2011), in the sense that the decrease in influx capacity largely overshadows the also decrease in efflux. The latter effect is simply unable to minimally compensate the collapse of the salvage system.

The additional possibility is that *pf*MRP1 also pumps out the anti-folate drugs themselves. *pfmrp1* deletion in NF54—followed by the ablation of the *hdhfr* selection marker with FLPe recombinase—does not significantly change the IC_50_s (72 h exposure, pLDH concentration based detection) for trimethroprim and WR99210 (Rijpma et al., 2016a). As for the anti-malarial pyrimethamine, a “small but significant” *increase* in sensitivity was reported (12–8 nM). Surprisingly, a 10 fold *increase* in methotrexate (MTX) IC_50_ response was documented in parallel (190–1800 nM). In a subsequent experiment, adding folate to the medium (10 fold, towards 23 nM) further increased the parasite resistance to MTX (less then 2 fold, IC_50_ = 2700 nM), attesting for the influence of the intracellular pool status (Rijpma et al., 2016a). Data concerning the other antifolate drugs were unfortunately not reported, albeit a similar de-sensitization outcome is expectable.

The specific effect on MTX upon *pfmrp1* ablation is left unanswered. This drug is a MRP substrate in eukaryotes, so the absence of the transporter should lead to its intra-parasitic accumulation, and a consequent decrease in IC_50_. MTX and folate accumulation experiments were not performed (at least the latter due to the small available sample volumes), but as referred IC_50_s were surprisingly increased. The interpretation of this data becomes somewhat difficult, opening the opportunity to engage in a radically different possibility. Can the MRPs act as an importer of both MTX and folate?

#### *Pfmrp1* and *pfmrp2* Gene Expression and Drug Response

Albeit *P. falciparum* genes have been considered as particularly inert in terms of response to external stimuli, there are indications that up-regulation can occur upon drug exposure (Silva et al., 2019). One difficulty of studying this response is the fact that drug pressure frequently leads to an almost immediate (clone dependent) slow down of the parasite intra-erythocytic cycle (Veiga et al., 2010). As the latter is tightly associated with specific patterns of gene expression, this becomes a confounding effect when attempting to distinguish the transcription specific effect attributed to antimalarial exposure. Upon filtering this effect through a non-linear regression model, exposure of 3D7, W2 and FCB clones to mefloquine (IC_50_, 48 h) drove a mild specific up to 1.5 fold increase in both *pfmrp1* and *pfmrp2* mRNA accumulation (Q-PCR, internal control: seryl-tRNA synthetase) (Veiga et al., 2010). In a parallel work, (Nogueira et al., 2010) detected large up-regulation responses for *pfmrp1* and *pfmrp2* (>>10 fold, Q-PCR, internal control: 18S sRNA) upon short-term (2 h) exposure of CQ at IC_50_ concentrations to 3D7 and Dd2 ring stages. It is conceivable that CQ might command a larger effect on *pfmrp1* and *pfmrp2* transcriptional behavior. Albeit this work further supports the hypothesis of drug-driven increases in transcriptional activity, it is to note that the effects of cycle delay were not considered, which might have magnified the actual response behavior.

A preliminarily study involving 48 h exposure to IC_50_ (112 µM) and IC_90_ (329 µM) levels of the alkaloid piperine (Q-PCR. Internal control: beta-actin) did not find changes in *pfmrp1* mRNA accumulation (Thiegusuk et al., 2018).

The previously described studies were not designed to appraise the drug response phenotypic consequences of increased expression. This has been elegantly approached for *pfmrp2* by identifying a 3D clone derivative with an extensive 4.1 kD deletion in the AT-ultra rich 5′proximal regulation region of this gene (Mok et al., 2014). This mutation (3D7-6A) led to a significant increase in *pfmrp2* transcript accumulation and consequent protein levels when compared to the wild type (referred as 11C). Further, the mutation changed the patterns of *pfmrp2* expression along the cycle, with a later peak, now at the trophozite and schizont stages. The 3D7-6A clone had a 1.5-2 fold increase in IC_50_s to MEF and CQ at these stages, but not when the drug treatment was initiated in the earlier ring stage. As for CQ, 3D7-6A trophozoites/schizonts reached IC_50_s (*ca*. 80 nM) near the normally associated with clinical resistance, albeit independent of verapamil sensitization action. Mild decreases in sensitivity to quinine and lumefantrine were also registered (1.2–1.3 fold). Luciferase based promoter expression reporter essays pointed for a 2-6 fold increase in activity associated with the mutant promoter, the authors suggesting that the deletion might have created a new DNA element able to support a more active transcription initiation. Also, the deletion shortened the distance between *pfmrp2* and the PFL1415w gene (in the opposite strand), with the former apparently “gaining” the latter transcription cycle pattern. This leads to the suggestion of the presence of a bidirectional promoter element between the two genes that became sufficiently proximal of *pfmrp2* to influence its cycle of expression.

In conclusion, these works demonstrated that increased *pf*MRP expression can lead to drug evasion, independent of changes in the ORF sequence.

### *pf*MRPs—Importers, Exporters, or Both?

The 3D structure of *pf*MRP1 and 2 have not been reveald, yet it is assumed that they likely resemble other eukaryotic MRPs (Wilkens, 2015). Bozdech and Ginsburg (2004) have made the early suggestion that the significantly earlier *pfmrp2* transcriptional peak—before the emergence of full functional Hb digestions and glutathione synthetase expression—could be related with very different functions, namely the possibility of *pf*MRP2 acting as an importer of reduced glutathione (GSH) from the RBC, compensating the low activity of the parasite glutathione metabolism during that period of the intra-RBC cycle. This is not an outlandish suggestion. Among eukaryotes, albeit ABC transporters are commonly substrate exporters, a number of importing ones have been described, mainly in evolutionarily more primitive plants (Choi and Ford, 2021), but also in higher mammals, with the human ABCA4 being a prime example (Quazi et al., 2012). In parallel, it is to note that the most well studied *P. falciparum* ABC transporter, the P- glycoprotein homologue (Pgh) is actually considered to act as an importer towards the food vacuole lúmen (Rohrbach et al., 2006), albeit it has the advantage of being inserted in an internal membrane, which permits the direct access to cytoplasmic ATP for the protein NBDs. Considering the present available data, the notorious functional flexibility of the ABCC proteins, and the fact that the NBDs of a *pf*MRP importer in the parasite plasma membrane is still not actually exposed to extracellular space, but rather to the parasitophorous vacuole lumen, this possibility should not be discarded.

Under the “importer hypothesis”, it is then possible that *pf*MRP1 might actually work as a secondary folate transporter *towards* the parasite, complementing the function of the characterized *pf*FT1/*pf*FT2 proteins (Salcedo-Sora et al., 2011). Such configuration could explain the selection of the *pf*MRP1 1466R SNP, now hypothesized to be related with a more efficient folate importer structure, increasing the intra-cellular competition between this compound and pyrimethamine accessing DHFR (Dahlstrom et al., 2009a)—one is anyway left wondering why this allele is not the dominant one in nature. It would also support the strong MTX decrease in sensitivity in the absence of *pf*MRP1, as this drug—structurally related with folate—would simply be less transported towards the parasite. Also, and as proposed for *pf*FT1/*pf*FT2, it would explain the sensitization effect of probenecid, a long known resistance reversal agent that also affects MRPs (Barrand et al., 1997). Intriguingly, the parasite does not become more resistant to the structurally less folate-like pyrimethamine. In fact, *pf*MRP1 absence slightly decreases PYR IC_50_s, as well as sensitivity (and intra-cellular accumulation) to quinolone antimalarials in W2 (Raj et al., 2009) (but not 3D7-like NF54), pointing for a drug exporter function. This is further supported by the decrease in GSH accumulation arguing for a typical MRP exporter activity, the MRP absence making the parasite more vulnerable to direct and indirect (REDOX stress) antimalarial action.

Concerning *pf*MRP2, and loosely following the Ginsburg/Bozdech suggestion, one could also argue that the disruption of the hepatic merozoite development through *pfmrp2* deletion (Rijpma et al., 2016) is related with a resulting lack of import of essential substrates (e.g. co-factors, as it happens with some bacterial ABC importers) obtained from the liver cell during that stage.

In conclusion, part of the available data could be explained by both MRPs working as importers and/or exporters, leading to the rather intriguing possibility of *P. falciparum* MRP system having at least two main alternative conformational states, associated with markedely different insertion structures in the membrane, and altered functional capacities.

## Expert Opinion

**FIGURE 2:**
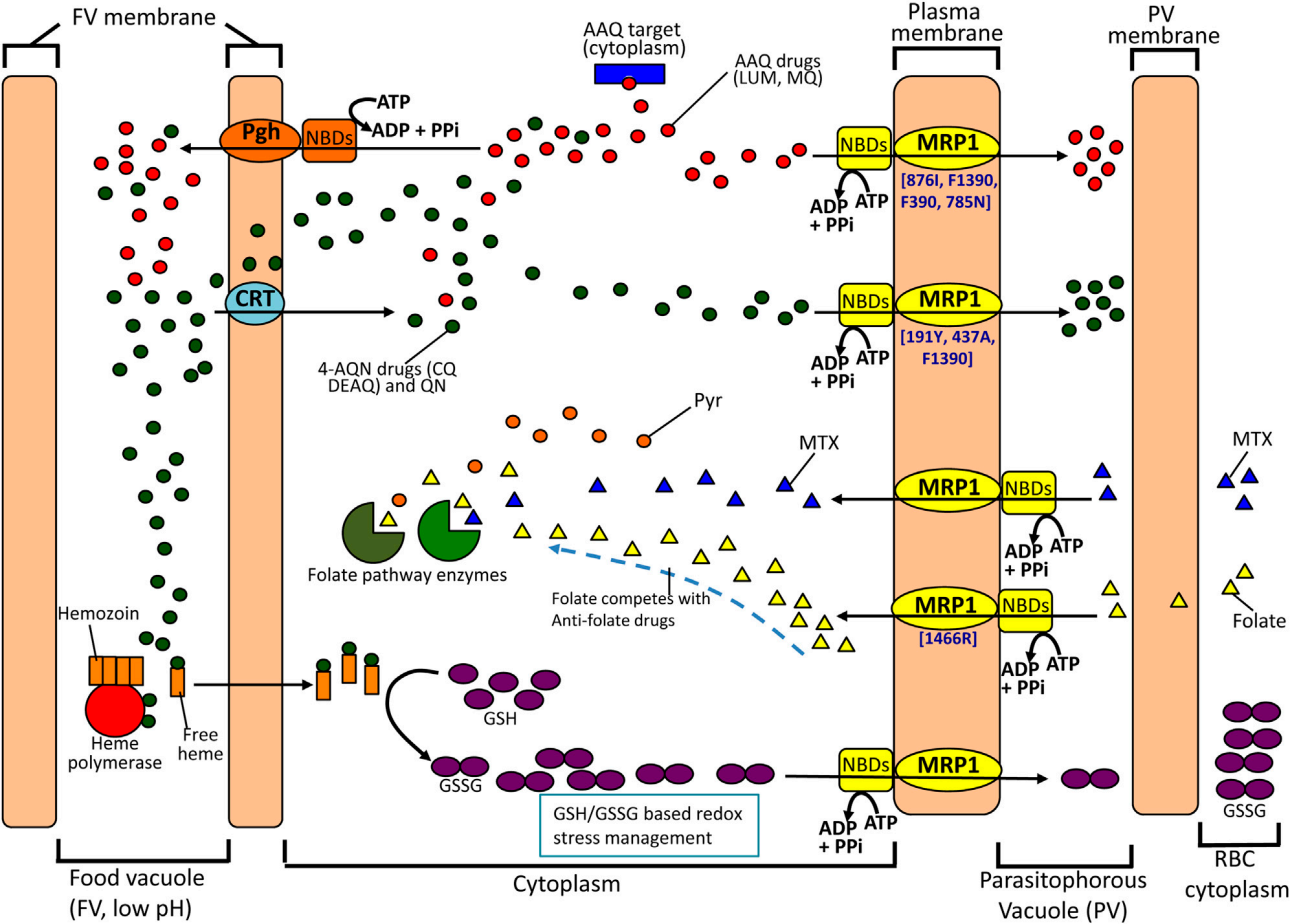
Possible contributions of *pf*MRP1 to drug resistance. *pf*MRP1 is essentially located in the parasite plasma membrane, putatively transporting antimalarial drugs. This action can be particularly important (and sufficient) concerning resistance to antimalarials which targets reside mainly in the cytosol or in the plasma membrane (e.g. mefloquine, that has been proposed to affect the phagocytosis of hemoglobin). Here, it might work in conjugation with Pgh, located in the food vacuole. For the more food vacuole centric antimalarials, namely 4-aminoquinolines (CQ, DEAQ), *pf*MRP1 might act as a second step in a two phase system of efflux. Also, resilience against antimalarials might include the role of *pf*MRP1 in the protective functions of the glutathione metabolism, through the efflux of its oxidized form (GSSG). This is expected to be important, as many antimalarials exert oxidative stress in the parasite, namely through increased levels of free heme. Finally, the *pf*MRP1 capacity of transporting folate is suggested to drive the involvement of this protein in antifolate resistance, by participating in the import of folate, which will directly compete with antifolate drugs for their target. Increases in intracellular pool will lead to less drug action. (LUM, lumefantrine; MQ, mefloquine; CQ, chloroquine; Pyr, pyrimethamine; AAQ, aminoalcohol quinolone; 4-AQN, 4-aminoquinoline; QN, quinine; MTX, methotrexate; Pgh, P-glycoprotein homologue; CRT, chlroquine resistance transporter).

20 years after its discovery, the involvement of *pf*MRPs on parasite antimalarial drug response is undeniable, as supported by a relatively robust number of studies. To celebrate this conclusion, a visual summary of the overall potential participation of *pf*MRP1 in particular is presented in Figure 2. Unfortunately, the real characteristics and clinical importance of these genes are still somewhat unclear. In part this is probably motivated from a likely frequent but secondary role in the phenomena, possibly being an often necessary, but not sufficient factor. Secondly, most studies available are *in vitro* based and small in size (<50 strains) and statistical power; also the *pfmrp* genes have been mostly analyzed for specific subsets of SNPs, when it is likely from the dispersed data that haplotype configurations must be key. Additionally, very few reports aimed on the *in vivo* context are available, namely the occurrence of treatment-driven polymorphism selection. Finally, the possibility of these transporters to work as both exporters and importers, if proven correct, will add a new layer of complexity for interpreting genotype-phenotype associations.

Times are also changing, which might propel the importance of *pf*MRPs. During this decade, a constellation of new anti-malarials will be launched, some in combination with old ones. The response of the parasite populations to this new wave of drug challenge is uncertain, albeit recent research points for ABC transporters to have a role (Murithi et al., 2021). It is reasonable to consider that *pf*MRPs, with their characteristic capacity of transporting a broad range of chemical structures, and gateway localization in the plasma membrane will have an important role. In parallel, the possibility to explore these transporters as drug targets themselves is open (Henry et al., 2008a; Daunes and D'Silva, 2018). In this scenario, the blockage of liver schizont formation is particularly attractive (Rijpma et al., 2016). It suggests the tantalizing prospect of drugs specifically targeting *pf*MRP2, leading to a novel class of liver stage-centric anti-malarials, an area with very few, and far from ideal, therapeutic options.

In conclusion, during the present decade we expect for research *pf*MRPs to take a more central stage, investigating its role in malaria drug resistance, and the possibility of these tranposrters being themselves druggable targets for next generation antimalarials.

## Footnotes

1
https://www.malariagen.net/apps/pf/4.0/#variation

2
https://www.malariagen.net/apps/pf/4.0/#variation
